# Application of Interactive Video Games as Rehabilitation Tools to Improve Postural Control and Risk of Falls in Prefrail Older Adults

**DOI:** 10.34133/2021/9841342

**Published:** 2021-06-25

**Authors:** Hammad S. Alhasan, Patrick C. Wheeler, Daniel T. P. Fong

**Affiliations:** ^1^National Centre for Sport and Exercise Medicine-East Midlands, School of Sport, Exercise and Health Sciences, Loughborough University, Loughborough, Leicestershire, UK; ^2^Physiotherapy Department, Faculty of Applied Medical Sciences, Umm Al-Qura University, Mecca, Saudi Arabia; ^3^Department of Sport & Exercise Medicine, University Hospitals of Leicester NHS Trust, Leicester, UK

## Abstract

The purpose of this study was to examine whether interactive video game (IVG) training is an effective way to improve postural control outcomes and decrease the risk of falls. A convenience sample of 12 prefrail older adults were recruited and divided into two groups: intervention group performed IVG training for 40 minutes, twice per week, for a total of 16 sessions. The control group received no intervention and continued their usual activity. Outcome measures were centre of pressure (COP), mean velocity, sway area, and sway path. Secondary outcomes were Berg Balance Scale, Timed Up and Go (TUG), Falls Efficacy Scale International (FES-I), and Activities-Specific Balance Confidence (ABC). Assessment was conducted with preintervention (week zero) and postintervention (week eight). The intervention group showed significant improvement in mean velocity, sway area, Berg Balance Scale, and TUG (*p* < 0.01) compared to the control group. However, no significant improvement was observed for sway path (*p* = 0.35), FES-I (*p* = 0.383), and ABC (*p* = 0.283). This study showed that IVG training led to significant improvements in postural control but not for risk of falls.

## 1. Introduction

Postural control is an essential human movement ability; optimum body posture and control of movement is a cornerstone of successful performance of activities of daily living, such as standing and walking [1]. However, postural control is affected by ageing due to visual, musculoskeletal, vestibular, somatosensory, and central nervous system alterations that lead to physical and cognitive impairments [2]. Postural control is a complex multidimensional process; to select the best motor strategies when balance is disturbed, postural control utilizes both feed-forward and feedback mechanisms [3, 4]. Feed-forward mechanisms are used to maintain postural control during voluntary movements or when anticipating a hazard, while feedback mechanisms are used when faced with sudden unexpected disturbance or when feed-forward fails [5]. It is anticipated that the use of biofeedback in the form of interactive video games (IVGs) could help increase participant's awareness of postural control.

The British Geriatrics Society defines frailty as a “distinctive health state related to the ageing process in which multiple body systems gradually lose their in-built reserves” [6]. It is a condition with different causes and contributors characterized by decreased strength, endurance, and physiological function, which together increase the vulnerability of older adults to becoming disabled [7]. Aerobic and strengthening exercises are recommended for both healthy and disabled older adults [7, 8]. According to Gill *et al*. [9], frailty is a reversible phenomenon that can be prevented or postponed if it is managed properly by valid and specifically designed interventions. Therefore, designing effective interventions to control or delay frailty is a public priority.

In the UK, the total number of adults aged over 65 in 2019 was 23% more than in 2009, with an increase of 2.3 million within 10 years, making them the age group with the highest level of growth [10]. Additionally, around 28% of adults aged over 50 reported being physically inactive, with approximately 10 million fall-related injuries reported and around 5 injuries per 100 people [11]. The prevalence of prefrailty in the community is about 53%, while the prevalence of frailty is about 17% [12]. Moreover, with the presence of chronic disease, it becomes more prevalent [13]. Thus, if an older adult individual is diagnosed as being frail, this means that he or she exhibits a greater risk of falling, low mobility, and disability [14].

UK Chief Medical Officers' physical activity guidelines recommend that older adults should engage in exercises that challenge postural control, which include resistance and balance training [15]. Additionally, evidence supports the use of multicomponent exercise to improve gait, postural control, and strength [16–20]. Multicomponent or combined exercise consists of aerobic, resistance, flexibility, and balance exercises [8]. A systematic review on the effectiveness of traditional exercise among frail older adults suggests that multicomponent exercise is an effective training for frail older adults; however, findings conclude that the optimal intervention in patients with frailty is unclear [21]. A previous systematic review highlighted inconsistent evidence of any effect of IVGs training on static postural control ability among frail older adults [22]. The lack of definitive guidance from these reviews could be due to the fact that they used different definitions for frailty which include nonfrail and prefrail individuals. Thus, due to no consensus on the definitive definition of frailty, some authors follow strict criteria that constitute frailty that does not include prefrail or nonfrail older adults with some disability, while others follow more flexible criteria. Additionally, lack of definitive guidance from these reviews could be due to the ceiling effect that exists with functional outcomes that use rating scores and performance time such as Berg Balance Scale and Timed Up and Go (TUG). The ceiling effect is seen when a significant number of participants achieve the highest score on a measurement [23]. The use of quantitative measurements, such as centre of pressure (COP) analysis, could provide additional information about postural control performance and overcome the ceiling effect found in functional outcomes [24, 25].

For the use of interactive video games, a systematic review reviewed seven studies that used IVGs to improve mobility and muscle strength; the findings from this review supported the use of IVGs and reported that they were feasible and acceptable for older adults [26]. For postural control and risk of falls, a recent review and meta-analysis of nine studies found that there was a significant effect on postural control functional outcomes but not on falls, but emphasized the need for standardisation of the training and criteria for confirming frailty [16]. Despite these findings, IVGs for postural control are still in their infancy that the optimal training program is not yet clear [27]. Furthermore, there is limited evidence of the effectiveness of IVGs in frail older adults [28]. Therefore, this study's primary focus was to examine whether eight weeks of IVG training is an effective way to improve postural control outcomes and decrease the risk of falls among frail and prefrail older adults. The IVG protocol consists of games focused on repetitive and voluntary movements in different directions, with varied speed and precision. The researchers hypothesised that the proposed training program would significantly improve objective (COP parameters), functional (Berg Balance Scale and TUG), and subjective (Activities-Specific Balance Confidence (ABC) Scale and Falls Efficacy Scale International (FES-I)) postural control outcomes.

## 2. Materials and Methods

This study was designed to investigate the use of IVG on postural control and the risk of falls in frail and prefrail older adults. In this trial, Nintendo Wii Fit Plus™ off-the-shelf games and the Wii balance board system were used as an IVG training tool.

### 2.1. Ethical Approval and Consent to Participate

Potential participants who met the inclusion criteria were informed of the study's purpose, and all the possible benefits and risks of participating were explained to them. Those interested in taking part were required to sign a consent form for the study procedure in accordance with the protocol approved by the Loughborough University Ethical Advisory Committee R19-P179.

### 2.2. Participants and Study Setting

All participants involved in the experimental study were recruited from Loughborough and nearby areas through community advertising, social media, a monthly online newsletter, the local neighbourhood, and community centres for older adults. All assessments and training sessions took place at Loughborough University. Evaluations were conducted preintervention (week zero) and postintervention (week eight). A summary of the participants' sociodemographic and clinical characteristics at baseline can be found in Table 1.

### 2.3. Eligibility Criteria

Participants who met all these criteria were considered eligible: frail and prefrail older adults aged 65+, scoring at least one out of ten in Frailty Index for Elders (FIFE) score of 1 − 3 = prefrailty and a score of ≥4 indicates frailty; this tool has strong content validity (range 0.50-1.0) [29], is able to walk independently and perform physical exercises in the orthostatic position, has good hearing acuity with normal or corrected visual acuity, and has no neurological disorders.

Participants were ineligible to take part in this study if they have absolute contraindications for exercise or any potential issue that might compromise safe study participation, have any physical, visual, or cognitive condition(s) that made it difficult for them to use the IVGs, experienced any falls secondary to syncope or acute illness, receiving other physical interventions at the same time, have no history of falls in the previous 12 months, and are unable to understand and communicate or use wheelchairs or walkers for mobility.

### 2.4. Data Collection

Participants were interviewed about their sociodemographic characteristics (age, gender, height, weight, and body mass index (BMI)), health (FIFE criteria, number of falls, and number of medications currently in use), and clinical conditions (postural control, gait, fear of falling, and risk of falling). All data were collected during a single, one-hour preassessment session.

### 2.5. Outcome Measures

Primary outcome measures were objective postural control assessment using a static posturography technique; the following COP parameters were computed: mean velocity, 95% confidence ellipse area or sway area, and sway path. Secondary outcome measures were Berg Balance Scale to assess the participants' ability or inability to safely balance during several predetermined tasks—assessing static postural control and the risk of falls. The TUG test was used to assess the participants' mobility and walking ability. FES-I was used to assess participants' fear of falling, and ABC was used to assess participants' confidence in performing activities of daily living. For detailed information about the assessment, refer to Appendix A.

### 2.6. COP Data Acquisition and Procedures

This study followed general recommendations for stabilometry research [30, 31]. Participants were evaluated while standing still for 60 seconds under two visual conditions (eyes open and eyes closed). Three trials with eyes open were conducted first, followed by three trials with the eyes closed. Throughout the assessment sessions, the participants were allowed to rest at any time until they felt capable of resuming. For detailed information about COP formula, calculation, and data collection, refer to Appendix B.

### 2.7. Intervention

The intervention comprised eight weeks of IVG exercises using the Nintendo Wii Fit Plus™. The intervention group performed this activity for 40 minutes, twice per week, for a total of 16 sessions. The members of the control group received no intervention and continued their usual daily activities. For a description of the games used, refer to Table 2. For more details about the intervention, refer to Appendix C.

### 2.8. Sample Size

To calculate the sample size for the current study, we used the statistical tool G∗power 3.1.9.7. The COP parameters were the primary outcomes in this study. We set the effect size to 0.45, with an alpha level of 5%, power of 80%, and ANOVA repeated measures between-within interaction. The calculation showed that the total sample size required to achieve sufficient power is 12.

### 2.9. Statistical Analyses

The Shapiro-Wilk tests were used to verify the normal distribution hypothesis. Sphericity was confirmed via the Mauchly test. The Student *t*-test or Mann-Whitney *U* tests were used to compare the intervention and control groups in terms of age, height, weight, and BMI. The results were considered significant when *p* < 0.05. Generalised effect sizes were calculated for all analyses. Note: Cohen's effect sizes (*r*) were defined as *r* < 0.20 = trivial; *r* < 0.21–0.50 = small; *r* < 0.51–0.80 = moderate; *r* ≤ 0.81 = large. The appropriate statistical test for the current dataset was a two-way mixed (between-within) analysis of variance (ANOVA). The two groups (IVG and control) made up the between-measures independent variables, whereas the phases of the training over time (pre- and posttraining) made up the within-measures independent variables. Statistical analysis was performed using SPSS Statistics 27.0 (IBM, Armonk, NY, USA). The data that support the findings of this study are available from the corresponding author Fong DTP upon reasonable request.

## 3. Results

The study recruited a convenience sample of 12 frail and prefrail older adults (8 females, 4 males); 6 of whom were placed in the intervention group (3 males, 3 females) and the other 6 in the control group (5 females, 1 male). Baseline data showed no significant difference between the two groups, and statistical analysis showed that both groups were comparable in terms of age, height, weight, BMI, and the number of falls.

All pre- and posteyes closed and eyes open sway area, mean velocity, and sway path data for both the intervention and control groups were normally distributed (Shapiro-Wilk *W*_(6)_ ≥ 0.755, *p* ≥ 0.022), and the mean variances were also homogeneous (Levene's *F*_(1, 10)_ ≤ 8.970, *p* ≥ 0.013). All data were measured at the ratio level. All pre- and post-TUG, Berg Balance Scale, ABC, and FES-I data for both groups were normally distributed (Shapiro-Wilk *W*_(6)_ ≥ 0.803, *p* ≥ 0.62), and the mean variances were also homogeneous (Levene's *F*_(1, 10)_ ≤ 2.782, *p* ≥ 0.126).

A significant group-by-time interaction was found for sway area and mean velocity in eyes open and eyes closed conditions, respectively (*F*_(1, 10)_ = 23.998, *p* < 0.01), (*F*_(1, 10)_ = 15.003, *p* < 0.01), (*F*_(1, 10)_ = 21.100, *p* < 0.01), and (*F*_(1, 10)_ = 43.526, *p* < 0.01), and for Berg Balance Scale (*F*_(1, 10)_ = 14.412, *p* < 0.01) and TUG (*F*_(1, 10)_ = 13.602, *p* < 0.01). Summary of all pre- and postassessment scores can be found in Table 3. For a detailed description of the results, refer to Appendix D.

## 4. Discussion

The results from the current study indicate that eight weeks of IVG training twice per week using Nintendo Wii Fit Plus™ is effective in improving postural control among frail and prefrail older adults. The main findings were a significant improvement in COP mean velocity and sway area in both eyes open and eyes closed conditions. Similarly, Berg Balance Scale and TUG were also significantly improved. However, no significant improvement was observed for sway path, FES-I, or ABC.

Maki *et al*. [32] [32] reported that most falls among older adults are due to inadequate control of one's sway when faced with perturbation; the inadequate control can be from increased or decreased sway. For COP parameters, the findings are in line with Cho *et al*. [33], who utilized 8 weeks of Wii training on older adults for 30 minutes, 3 times per week, and found there was a significant reduction in COP sway area in eyes open and eyes closed after the training compared to the control group. Although they used the same IVG device, they utilized different games, mainly balance games. Additionally, Schwenk *et al.* [34] found that four weeks of IVG training using wearable sensors showed a significant reduction in COP sway area compared to no training. Similarly, Sihvonen *et al*. [35]) reported that the most observed improvement was in sway area. In contrast, Jorgensen *et al*. [36] reported no significant reduction in mean velocity after 10 weeks of Wii training. A potential reason is that they recruited healthy, highly functioning participants, which limits the generalizability of their results. Overall, a recent meta-analysis by Pacheco *et al*. [37] showed that IVGs yield a significant effect on COP sway (SMD = −0.89; 95%CI = −1.26 to − 0.51; *p* = 0.0001; *I*^2^ = 58%). It is worth noting that there is serious heterogeneity in this data; thus, it should be interpreted with caution.

There was a reduction but not significant in sway path in the IVG group. This finding is similar to previous studies that found that when training was conducted on a fixed surface, it did not yield significant changes in sway path, as it is easier than training on a moving surface (38, 39). In contrast, Schwenk *et al.* [34] found that using wearable sensors showed significant improvement in COP sway compared to no intervention. They attributed the improvement to the use of obstacle training concluded that excluding dynamic stepping exercises may result in no improvement in sway path. Wearable sensor training has the advantage of using virtual obstacles over platform-based training; the use of virtual obstacles overcomes the hazards associated with physical obstacles such as falling; this is the main reason why physical obstacles are not used with frail population [34]. A recent review found that most IVG studies did not involve dynamic stability training [38]. There is no known commercial IVG device that has this feature. However, the stepping game used in our training could potentially serve this purpose, as the participants were asked to step on and off the balance board repeatedly.

Our findings support the notion that visual feedback can help improve postural control [35]. Recent randomised control trial RCT by Sadeghigei et al. [39] found that IVG training yielded significant improvement in postural control and functional mobility compared to traditional training and control. Overall, their conclusion was to use a combination of IVGs and traditional training to obtain the best results. Although they recruited healthy older adults, their findings were similar to ours. The improvement in postural control for participants could be due to the nature of the IVGs used, as IVGs have the advantages of auditory and visual feedback, along with enjoyment and competition. Additionally, IVGs combine physical and cognitive training, require greater sensory integration, and focus on multidirectional movement, weight shifting, planning, and decision-making [40–42]. Our protocol focuses on educating participants about their sway and having them control it in various situations. Most games used challenged participants to maintain their COP as steady as possible. This will help improve their feed-forward strategy, which is achieved by controlling their avatars on the screen while they play. We hypothesised that the training would have a positive effect on all outcomes due to the nature of the games, as it challenged participants' postural control by having them move rapidly in different directions and shift their COP. All of these advantages make IVG training more challenging than other forms of exercises and maximize the effectiveness of the training.

Our TUG results contradict findings from Liao *et al*. [43], who found that IVG training using Microsoft Kinect yields no significant effect when compared to combined exercises on TUG and FES-I. Participants in their study were recruited from older adults care centres where training is offered to residents, which can be a serious confounding factor; additionally, the IVG group participants were younger than their peers in the combined exercise group. Similarly, Karssemeijer *et al*. [44] found no significant difference between the IVG, control, and aerobic exercise groups on TUG. Their participants were diagnosed with dementia, which limits the generalizability of their findings. On the other hand, Jorgensen *et al*. [36] concluded that 10 weeks of Wii training showed a significant reduction in TUG compared to wearing copolymer insoles. This study utilized Wii training similar to ours. The improvement in TUG can be interpreted as an improvement in frailty status. The British Geriatrics Society guidelines mention that the use of the TUG test is an indicator of frailty with a cut-off score of more than 10 seconds [6].

When reviewing the literature concerning IVGs and older adults, most studies focused on the use of functional and subjective outcomes; rarely was a force plate or a biomechanical method of assessment reported [16]. Functional assessments such as Berg Balance Scale have a ceiling effect and can be evident in the healthy older adults, which is an unavoidable limitation [45]. Additionally, the cut-off score for the Berg Balance Scale was not consistent among studies ranging from 45 to 33 points [46–48]. For subjective outcomes, asking participants about their history of falls is the most important predictor of future falls [49]. This can be biased by the patient if he or she has cognitive impairments, resulting in inaccurate data. Additionally, it may be biased by participants' psychosocial status, meaning that their physical capacity could be perfect, but factors such as fear of falling are interfering and affecting their confidence. Our findings showed that participants' subjective measures did not change significantly, while the objective measures did. There is evidence that supports the use of COP parameters, such as mean velocity and sway area, as predictors of future falls, especially during eyes closed conditions [32, 50–52].

### 4.1. Limitations

This study did not compare IVGs to other forms of training, so future studies with active control should be carried out to ensure that the improvement was from the IVG itself. There was a lack of long-term follow-up, as most participants were lost or unable to continue due to COVID-19. This led to a small sample size, which limited the generalizability of the results. Future studies could offer a longer period of training with a longer follow-up to confirm whether the effect was retained. Participants were not blinded to the training, as this was an uncontrollable limitation. For safety and monitoring purposes, we were unable to blind the assessors.

In this study, we focused on the use of static posturography. Knowing that IVG training was dynamic in nature, the use of a proper dynamic posturography assessment could provide deep insight into postural control improvement. Therefore, future studies should focus on this aspect. For outcomes that did not improve significantly, it can be argued that frailty management is multifaceted, meaning that it requires care from a team of healthcare providers [53]. In our study, we focused solely on the physical aspect, neglecting other aspects, such as nutrition, which could affect the result.

## 5. Conclusion

This study showed that eight weeks of IVG training twice a week, involving multicomponent exercises for frail older adults, led to significant improvements in COP sway area, mean velocity, TUG, and Berg Balance Scale. However, ABC and FES-I remained unchanged. These findings support the use of IVGs in frail populations.

## Figures and Tables

**Table 1:** Summary of the participants' sociodemographic and clinical characteristics at baseline (data are presented as the mean ± SD).

Parameter	Intervention group (*n* = 6)	Control group (*n* = 6)	*p* value
Age (years)	70.5 ± 5.5	72 ± 5.9	0.90
Height (cm)	172 ± 6.2	166.8 ± 4.9	0.48
Weight (kg)	82 ± 12.3	69.6 ± 10.1	0.52
BMI (kg/cm^2^)	29.2 ± 3.3	24.9 ± 2.4	0.50
Frailty Index for Elders (FIFE) score	1.6 ± 0.08	2.1 ± 1.1	0.48
Number of falls in the previous year	2.8 ± 1.9	2.3 ± 1.2	0.31

**Table 2:** Games description.

Game	Description
Basic Step	Participants were asked to step on and off the balance board, following the rhythm presented on the screen.
Balance Bubble Plus	Participants shifted their weight on the balance board in all directions to steer a bubble through obstacles.
Rowing Squat	Participants performed mini squats on the balance board with elbow flexion and shoulder adduction.
Soccer Heading	Participants shifted their weight right and left to hit a coming ball and avoid other objects.
Half-Moon	Participants raised their arms, bent their hips left and right, and tried to maintain this pose.

**Table 3:** Summary of all assessment scores. Data are presented as the mean ± SD.

Outcome measure	Intervention group (*N* = 6)		Control group (*N* = 6)		Time × group, *p* value
	Preintervention	Postintervention	Preintervention	Postintervention	
*Eyes open*					
Sway area (cm^2^)	2.8 ± 0.8	1.9 ± 0.4	1.4 ± 0.5	2.5 ± 0.3	*p* < 0.001
Mean velocity (cm/s)	1.9 ± 0.5	1.2 ± 0.1	1.2 ± 0.1	1.7 ± 0.3	*p* < 0.001
Sway path (cm)	2.9 ± 1.3	1.5 ± 0.4	2.1 ± 0.6	3.1 ± 0.7	*p* = 0.078
*Eyes closed*					
Sway area (cm^2^)	3.7 ± 1.6	2.5 ± 1.1	3.2 ± 1.7	4.1 ± 2.0	*p* < 0.001
Mean velocity (cm/s)	2.1 ± 0.5	1.4 ± 0.3	1.4 ± 0.2	1.9 ± 0.3	*p* < 0.001
Sway path (cm)	3.4 ± 1.0	2.2 ± 0.7	2.0 ± 0.9	3.8 ± 1.8	*p* = 0.350
Berg Balance Scale	40 ± 2.8	42 ± 3.2	41.5 ± 2.5	41.1 ± 2.0	*p* < 0.001
TUG (seconds)	15.1 ± 2.4	14.5 ± 2.4	13.9 ± 1.6	14.9 ± 1.2	*p* < 0.001
FES-I	29.3 ± 7.1	29.1 ± 5.8	28.8 ± 4.3	29.5 ± 3.5	*p* = 0.280
ABC	78.2 ± 4.8	79.1 ± 4.5	77.7 ± 3.2	77.8 ± 3.2	*p* = 0.380

**Table 4:** MATLAB codes for COP parameters.

COP parameter	MATLAB code
Sway path	sway path=sum(sqrt(CPap.^2+CPml.^2)).
Mean velocity	mean velocity=sum(sqrt(diff(CPap).^2+diff(CPml).^2))∗freq/length(CPap).
Sway area or 95% prediction ellipse area	[vec,val]=eig(cov(CPap,CPml)); Area=pi∗prod(2.4478∗sqrt(svd(val))).

## Data Availability

The data that support the findings of this study are available from the corresponding author Fong DTP upon reasonable request.

## Appendix

### A. Methods

#### A.1. Methods

Two trained researchers evaluated all participants at two time points, immediately before and after the training.

#### A.2. General Assessment

1. The participants were required to fill out a Health Screen Questionnaire for Survey Volunteers. This was done to ensure that the participants were in good health and that they had had no significant medical problems, as well as to ensure their continued well-being and avoid the possibility of individual health issues confounding the study outcomes
2. To assess their frailty risk, participants were asked to fill out FIFE forms. All participants were at risk of becoming frail, except for one participant, who was already frail

#### A.3. Subjective Postural Control Assessment

1. To record their histories of falls, participants were asked the following questions: “Did you slip, trip or fall in the last year? If yes, how many times?” These questions were adopted from the National Institute for Health and Care Excellence (NICE) guidelines for assessing and preventing falls in older adults [54]. A fall in this study was defined as “any event that caused involuntary contact by the torso or upper limbs to the ground or to a lower level, other than as a consequence of a violent blow, loss of consciousness, or a sudden onset of paralysis as in stroke or epileptic seizure” [55]. A “faller” was defined in this study as a subject who had sustained more than one fall within the last 12 months
2. To assess functional mobility, participants were asked to rate their levels of confidence (0% to 100%) in performing various activities on the ABC Scale, which measures fear of falling and confidence in maintaining balance; they rated their confidence in performing 16 ADLs. Test-retest reliability of ABC is 0.92 with Cronbach's alpha of 0.96 [56]
3. To assess fear of falling, participants were asked to fill out the FES-I. It has an excellent test-retest reliability among community dwelling older adults (interclass correlation coefficient [ICC] = 0.96) [57]

#### A.4. Functional Postural Control Assessment

1. To assess postural control and risk of falls, the Berg Balance Scale [58] was employed, The Berg Balance Scale has a high intra- and interrater reliability (ICC = 0.98) [59], a 94.4% sensitivity, and a 54.8% specificity [60]
2. Finally, the TUG test [61] was used to assess mobility and dynamic postural control. Participants had to stand up from a chair walk at their normal pace 3 meters turn and return to the chair and sit down. The chair had armrests and the seat was 46 cm high. We used a stopwatch to calculate the time taken to finish the test timing start from the instruction “go” and stops when participants are seated. We recorded three trials for each participant then we take the average

#### A.5. Objective Postural Control Assessment

In this study, objective postural control assessments were made using static posturography via traditional postural sway measurements. Traditional COP parameters were used to describe the area, distance, and velocity of the COP trajectory.

### B. COP Data Analysis

#### B.1. COP Formula and Calculation

The following calculation methods were adopted from Vieira *et al.* [62]:

COP sway path, or total excursion, is defined as “the length of COP trajectory on the BOS.” As the COP signal does not provide direct changes in postural control, sway path can be calculated by summing the actual distance between successive COP locations [63]. However, sway path's usefulness for quantifying changes in postural control is limited, and it must be supported by other COP parameters [64].

COP mean velocity is defined as “the total distance travelled by the COP over time.” It can be calculated by dividing the sway path by the trial duration, and it shows a high reliability when a double-legged stance is used (*R* = 0.84) [65].

COP sway area or 95% confidence ellipse area was calculated by computing 95% confidence ellipse of COP AP and COP ML coordinates. It is defined as “estimates the dispersion of the CP data through the calculus of the statokinesigram area” [66]. In other words, it contains 95% of the COP samples in 60 seconds. Sway area was calculated using principal component analysis to estimate its axes [67, 68].

In this research, the COP signals were processed on a computer connected to the ADC, using BioWare® software to obtain the COP traces. During the trials, the COP position traces were measured using a static force platform (Kistler®). For all COP data collected during bipedal quiet standing, a single force platform was used, with dimensions of 60 cm × 90 cm (42 cm × 70 cm between sensors). Data were sampled and collected at 100 Hz.

Raw COP data were processed using an automatic code written in MATLAB (MATLAB, version R2019b, Usway area). The MATLAB codes for calculating these parameters can be found in Table 4 and were adopted from Prieto *et al*. [69] and Březina [70]. An increase in COP parameters indicates inadequate postural control, while a decrease signals better postural control. The variables analysed were sway area, mean velocity, and sway path in the AP and ML directions. All parameters were computed for each trial and then averaged.

These parameters were chosen because they have been widely reported to be representative measures [71] and are highly correlated with falls in older adults [32, 72–74]. They also have a high interrater and test-retest reliability, with ICCs ranging from 0.70 to 0.89 [75]. Mean velocity was used in this study as it has been reported to be the most discriminative COP parameter for assessing age group differences concerning postural control and risk of falls [76, 77]. Sway area was chosen because previous researchers have found that using mean velocity and sway area together provides discriminative data between those with impaired postural control and those with no impairments, while the use of sway area alone does not allow for such discrimination [78].

The following steps were used for force platform data collection:

1. The force platform was zeroed according to the equipment's manuals
2. The researchers explained the data collection process to the participants. They were told that they would be monitored during the tests and that there should be no verbal communication during the trials, but they could interrupt the testing if necessary
3. The researchers measured the participants' body mass
4. The researchers asked the participants to maintain their posture as still as possible, with their arms at their sides
5. Data collection began three seconds after the participants said they were ready
6. At the end of each trial, the subject was given a one-minute rest period before the next trial commenced

### C. Intervention

#### C.1. Intervention

The training protocol was hypothesised and informed by the systematic review published earlier [16]. The intervention, based on the Nintendo Wii Fit Plus™, included cardiovascular, strengthening, balancing, and stretching exercises (multicomponent exercise). This was in line with recent evidence that favours such a multicomponent approach over traditional balance-only exercise [79]. Multicomponent exercise is defined as a “combined program of endurance, strength, coordination, balance, and flexibility exercises that have the potential to impact a variety of functional performance measures” [80].

All sessions were conducted individually. The Wii setup consisted of a balance board shaped like a weighing scale, measuring 8.5 in.×6 in.×2 in. This board was wireless (running on two AA batteries), which could support up to 135 kg and contained multiple pressure sensors that measured participants' COP and BMI. The IVGs were delivered on a 50-inch TV monitor, and participants stood 2 m away from the monitor.

#### C.2. Training Procedure

The training began with a warm-up game (“Basic Step”), in which the participants stepped on and off the balance board following a rhythm shown on the screen. Then, two balance games were administered (“Soccer Heading” and “Balance Bubble Plus”); both of which were designed to challenge the participants' balance abilities by asking them to shift their body weight in different directions (AP and ML). A strengthening game was placed between the two balance games, focusing on strengthening the quadriceps. In this strengthening game, the participants had to perform 15 to 30 mini squat repetitions. Finally, the sessions finished with a stretching exercise for the back and leg muscles (“Half-Moon”).

Each game lasted approximately two to three minutes, but the allotted session times were ten minutes for “Bubble Balance Plus” and five minutes for “Basic Step,” “Rowing Squat,” “Soccer Heading,” and “Half-Moon.” Thus, the participants played each game repeatedly until they reached the allotted session times. It took one to two minutes to change from one game to the next, and, during that period, the participants sat on a chair to rest.

### D. Results

#### D.1. Result

All participants had history of falls in the previous year, and mean number of falls was 2.5 ± 1.9 and their mean frailty index was 1.9 ± 0.99 (minimum = 1; maximum = 4), which indicate they were at risk of developing frailty. Participants in this study were deemed prefrail according to FIFE; to meet the definition of prefrail, they had to have a score of 1 to 3. The mean ABC was 78.9 ± 4.2 indicating they have moderate level of physical functioning. The mean FES-I was 26.3 ± 6.4, which indicates the samples are at moderate risk of falls. The mean Berg Balance Scale was 41.8 ± 2.8 indicating they are independent. Finally, the mean TUG was 13.1 ± 1.8 seconds indicating they are at risk of falls. Interpretation of all these scores indicates that they were at risk of having falls and inadequate postural control.

#### D.2. Objective Outcomes

*Eyes Open Sway Area*. A significant group by time interaction (*F*_(1, 10)_ = 23.998, *p* = 0.001) showed that the intervention group experienced a reduction in COP sway area over the training period (2.86 vs. 1.91 cm^2^) and effect size was large (Cohen's *d* = ∣1.09∣), whereas the control group's mean sway area was increased over the same period (1.45 vs. 2.51 cm^2^) and effect size was large (Cohen's *d* = ∣1.2∣).

*Eyes Open Mean Velocity*. A significant group by time interaction (*F*_(1, 10)_ = 21.100, *p* = 0.001) showed that the intervention group experienced a reduction in COP mean velocity over the training period (1.97 vs. 1.27 cm/s) and effect size was large (Cohen's *d* = ∣1.39∣), whereas the control group's mean COP mean velocity was increased over the same period (1.20 vs. 1.71 cm/s) and effect size was large (Cohen's *d* = ∣1.01∣).

*Eyes Open Sway Path*. A nonsignificant group by time interaction (*F*_(1, 10)_ = 3.91, *p* = 0.076) showed that the intervention group experienced a reduction in COP sway path over the training period (2.82 vs. 1.6 cm/s) and effect size was moderate (Cohen's *d* = ∣0.78∣), whereas the control group's mean COP sway path was increased marginally over the same period (2.59 vs. 2.78 cm/s) and effect size was trivial (Cohen's *d* = ∣0.12∣).

*Eyes Closed Sway Area*. A significant group by time interaction (*F*_(1, 10)_ = 15.003, *p* = 0.003) showed that the intervention group experienced a reduction in COP sway area over the training period (3.74 vs. 2.59 cm2) and effect size was moderate (Cohen's *d* = ∣0.67∣.), whereas the control group's mean sway area was increased meaningfully over the same period (3.23 vs. 4.12 cm^2^) and effect size was moderate (Cohen's *d* = ∣0.52∣).

*Eyes Closed Mean Velocity*. A significant group by time interaction (*F*_(1, 10)_ = 43.526, *p* ≤ 0.0001) showed that the intervention group experienced a reduction in COP mean velocity over the training period (2.18 vs. 1.46 cm/s) and effect size was large (Cohen's *d* = ∣1.31∣), whereas the control group's mean COP mean velocity was increased over the same period (1.39 vs. 1.95 cm/s) and effect size was large (Cohen's *d* = ∣1.01∣).

*Eyes Closed Sway Path*. A nonsignificant group by time interaction (*F*_(1, 10)_ = 0.932, *p* = 0.357) showed that the intervention group had a relatively stable COP sway path over the training period (2.48 vs. 2.68 cm/s) and effect size was small (Cohen's *d* = ∣0.23∣), whereas the control group's mean COP sway path was increased over the same period (2.58 vs. 4.35 cm/s) and effect size was large (Cohen's *d* = ∣2.19∣).

#### D.3. Functional Outcomes

*TUG*. A significant group by time interaction (*F*_(1, 10)_ = 13.602, *p* = 0.004) showed that the intervention group experienced a reduction in TUG over the training period (15.1 vs. 14.5 seconds) and effect size was small (Cohen's *d* = ∣0.23∣), whereas the control group's mean TUG was increased over the same period (13.9 vs. 14.9 seconds) and effect size was small (Cohen's *d* = ∣0.4∣).

*Berg Balance Scale*. A significant group by time interaction (*F*_(1, 10)_ = 14.412, *p* = 0.004) showed that the intervention group experienced an increase in Berg Balance Scale over the training period (40 vs. 42 points) and effect size was moderate (Cohen's *d* = ∣0.71∣), whereas the control group's mean Berg Balance Scale was decreased marginally over the same period (41.5 vs. 41) and effect size was small (Cohen's *d* = ∣0.11∣).

#### D.4. Subjective Outcomes

*ABC*. A nonsignificant group by time interaction (*F*_(1, 10)_ = 0.832, *p* = 0.383) showed that the intervention group experienced an increase in ABC over the training period (78.1 vs. 79.1 points) and effect size was trivial (Cohen's *d* = ∣0.18∣), whereas the control group's mean Berg Balance Scale was relatively stable over the same period (77.7 vs. 77.8) and effect size was small (Cohen's *d* = ∣0.01∣).

*FES-I*. A nonsignificant group by time interaction (*F*_(1, 10)_ = 1.289, *p* = 0.283) showed that the intervention group had almost the same FES-I score over the training period (29.3 vs. 29.1 points) and effect size was trivial (Cohen's *d* = ∣0.02∣), whereas the control group's mean Berg Balance Scale was increased marginally over the same period (28.8 vs. 29.5) and effect size was small (Cohen's *d* = ∣0.09∣).
